# 

**Published:** 1888-10

**Authors:** 


### CONTENTS OF THIS NUMBER.

**Original Communications—** PAGE.

Some Practical Deductions Drawn from the Known Functions of the Liver. G. W. Webster ... 193
The Primary Physiological Purpose of the Membrana Tympani. S. O. Richey ... 197
A New Method of Feeding in Cases of Intubation of the Larynx. W. E. Casselberry ... 201

**Editorial—**

Surgery of the Brain ... 203
The Outbreak of Yellow Fever ... 204
The Congress of American Physicians and Surgeons ... 204

**Society Reports—**

Congress of American Physicians and Surgeons ... 205
Association of American Physicians ... 213
American Surgical Association ... 219
American Gynæcological Society ... 230
American Otological Society ... 232
American Ophthalmological Society ... 234
American Orthopedic Association ... 235
American Rhinological Association ... 240
International Otological Congress ... 242
Chicago Medical Society—Stated Meeting, July 2 ... 243
Gynæcological Society of Chicago—Regular Meeting, June 29 ... 248

**Domestic Correspondence** ... 255
**Extracts and Abstracts** ... 255
**Announcements** ... 256

